# Support Methodologies for African American Women With Lupus – Comparing Three Methods’ Effects on Patient Activation and Coping

**DOI:** 10.3389/fpsyg.2021.734390

**Published:** 2021-10-05

**Authors:** Ashley White, Trevor D. Faith, Aissatou Ba, Aundrea Loftley, Viswanathan Ramakrishnan, Hetlena Johnson, Jillian Rose, Clara L. Dismuke-Greer, Jim C. Oates, Leonard E. Egede, Edith M. Williams

**Affiliations:** ^1^Department of Public Health Sciences, Medical University of South Carolina, Charleston, SC, United States; ^2^Biomedical Informatics Center, Medical University of South Carolina, Charleston, SC, United States; ^3^Division of Endocrinology and Diabetes, Medical University of South Carolina, Charleston, SC, United States; ^4^LupusCSC, Columbia, SC, United States; ^5^Community Engagement, Diversity and Research, Department of Social Work Programs, Hospital for Special Surgery, New York, NY, United States; ^6^Health Economics Resource Center, VA Palo Alto Health Care System, Menlo Park, CA, United States; ^7^Division of Rheumatology, Department of Medicine, Medical University of South Carolina, Charleston, SC, United States; ^8^Division of General Internal Medicine, Center for Patient Care and Outcomes Research, Medical College of Wisconsin, Milwaukee, WI, United States

**Keywords:** systemic lupus erythematosus, African American, women, patient activation, research methods

## Abstract

**Introduction:** Systemic lupus erythematosus (SLE) is a chronic inflammatory disease in which the immune system attacks healthy tissues. While pharmaceutical therapies are an important part of disease management, behavioral interventions have been implemented to increase patients’ disease self-management skills, provide social support, and encourage patients to take a more active role in their care.

**Methods:** Three interventions are considered in this study; peer-to-peer methodology, patient support group, and a patient navigator program that were implemented among largely African American women with SLE at the Medical University of South Carolina (MUSC). Outcomes of interest were patient activation and lupus self-efficacy. We used a Least Squares Means model to analyze change in total patient activation and lupus self-efficacy independently in each cohort. We adjusted for demographic variables of age, education, income, employment, and insurance.

**Results:** In both unadjusted and adjusted models for patient activation, there were no statistically significant differences among the three intervention methodologies when comparing changes from baseline to post intervention. Differences in total coping score from baseline to post intervention in the patient navigator group (−101.23, *p*-value 0.04) and differences in scores comparing the patient navigator with the support group were statistically significant (116.96, *p*-value 0.038). However, only the difference in total coping from baseline to post intervention for the patient navigator program remained statistically significant (−98.78, *p*-value 0.04) in the adjusted model.

**Conclusion:** Tailored interventions are a critical pathway toward improving disease self-management among SLE patients. Interventions should consider including patient navigation because this method was shown to be superior in improving self-efficacy (coping scores).

## Introduction

Managing systemic lupus erythematosus (SLE) can be a difficult process for patients and providers alike due to the complex nature of the disease. Clinical manifestation and severity can vary significantly between patients with some experiencing a milder disease course while others may experience organ involvement and frequent flares of symptoms ranging from fatigue, photosensitivity, and joint pain to neurologic impairments, inflammation of the kidneys (nephritis), and inflammation of the pericardium (pericarditis) (Tsokos, 2011). As a result, clinical supervision of the disease is critical for disease management and averting adverse outcomes. Medication regimens are often tailored across a multidisciplinary team of physicians for tolerability and each patient’s disease presentation (Van Vollenhoven et al., 2014).

However, racial disparities have been observed regarding treatment response and long-term disease activity level in patients with SLE. Non-white individuals are affected by the disease at greater rates with African American women having a 3–9 times greater risk of developing the disease (Pons-Estel et al., 2017). Moreover, the disease is more severe among African American women when compared to other racial groups, with end stage organ involvement, depression and symptom flares frequently observed (Pons-Estel et al., 2017; Jordan et al., 2019). The Lupus Low Disease Activity State (LLDAS) has been validated as a predictor of organ damage and mortality (Franklyn et al., 2016). Achieving a LLDAS-50 of greater than or equal to 50% has been shown to reduce mortality and organ damage (Franklyn et al., 2016). Babaoglu et al. (2019), investigated predictors of being in LLDAS ≥ 50% of the defined treatment time in a cohort of Caucasian and African American women with SLE. The study found that African American women were less likely to achieve LLDAS ≥ 50% despite adjustment for socioeconomic, serological and treatment variables. These data are compelling and indicate the need to investigate other treatment approaches, such as socio-behavioral interventions, that may be predictors of positive disease-response (Franklyn et al., 2016; Babaoglu et al., 2019).

Socio-behavioral interventions have been shown to impart improvements in coping and perceived social support among patients (Jordan et al., 2019). For example, patients who experience decreased physical function, stress and stigma associated with lupus can interact with their peers, share similarities and find solutions together (Giffords, 2003). This is important because the perception of self-management skills is associated with patient activation (Fortin et al., 2020). Such skills and an activated patient – one who is engaged in their care and health care decision making – improve outcomes and reduce disease activity among SLE patients (De Achaval and Suarez-Almazor, 2010). Furthermore, interventions targeting coping, support, and patient activation have been more frequently examined in literature as supplemental approaches to provide comprehensive disease management. These initiatives have utilized a variety of delivery methods including peer-to-peer, lay professional delivery, and healthcare professional mediated programs with varying levels of success in reducing disease activity, improving psychological symptoms, and increasing patient activation (Braden et al., 1993; Peterson et al., 1993; Maisiak et al., 1996; Edworthy et al., 2003; Sohng, 2003; Karlson et al., 2004; Lorig et al., 2004; Goodman et al., 2005; Ng and Chan, 2007; Navarrete et al., 2010; Drenkard et al., 2012; Balasubramanian et al., 2014; Lee et al., 2014; Williams et al., 2014, 2018). Therefore, a methodologic comparison of socio-behavioral interventions for African American SLE patients is warranted to determine if a specific delivery method is associated with increased self-efficacy or coping and patient activation. As such, the objective of this study was to compare three socio-behavioral intervention delivery methods: Peer-to-Peer, patient group support and patient navigator and examine their respective impact on coping and patient activation.

## Materials and Methods

Two SLE studies were used to compare three patient intervention methodologies. The peer approaches to lupus self-management (PALS) study examined an innovative, manualized peer mentorship program designed to provide modeling and reinforcement by peers (mentors) to other African American women with SLE (mentees) to encourage them to engage in activities that promote disease self-management. The care-coordination approach to learning lupus self-management (CALLS) study examined the effectiveness of a lay patient navigator for patients with SLE to improve disease self-management and quality of life and decrease indicators of disease activity. Peer-to-peer pairings and a patient support group were evaluated in the PALS study and a patient navigator mediated program was evaluated in the CALLS study. In the PALS study, the peer-to-peer pairings were the intervention group while the patient support group was the control. In the CALLS study, the patient navigator mediated program was the intervention group while the control group did not participate in an intervention. The intervention and control groups both continued to receive standard medical care. Both studies were implemented for patients with SLE at the Medical University of South Carolina (MUSC) and participants were largely African American women. While the PALS study had 120 participants with 100 of those used in these analyses, the CALLS study had 30, with only 14 of those eligible for these analyses. All participants were recruited at MUSC with the CALLS study being limited to patients with recent hospitalizations. Both studies used an active intervention period, or control follow-up period, of 12-weeks.

Using previously validated survey instruments implemented in both studies, the outcomes of interest for this methodology comparison were patient activation and coping measured as Lupus Self-Efficacy Scores. The patient activation measure (PAM) assesses an individual’s knowledge, skill, and confidence for managing their health and healthcare. Individuals who measure high on this assessment typically understand the importance of taking a pro-active role in managing their health and have the skills and confidence to do so (Hibbard et al., 2004, 2005). The patient activation total score was a composite of 10 questions from the patient activation measure and each question was assessed using a 4-point Likert scale. Coping was assessed by the Arthritis Self-Efficacy Scale pain and other symptoms sub-scale (Lorig et al., 1989), which consists of 11 items designed to measure confidence in one’s ability to manage the pain, fatigue, frustration, and other aspects of disease; it was reworded in previous investigations to reflect lupus rather than arthritis (Greco et al., 2004). The lupus self-efficacy score was the total coping score from the Lupus Self-Efficacy Scale and was assessed from six questions using a number scale of 1–10 (see attached instrument). In both studies these data were collected at baseline, mid-intervention (6 weeks from baseline) and post-intervention (12 weeks from baseline), and therefore comparable. Data were then organized and analyzed using SAS 9.4.

We ran unadjusted and adjusted linear mixed models for patient activation total score and the total coping score as separate outcome variables to look at changes from baseline to post intervention for each intervention methodology. Methodology group (patient navigator, support group, or peer-to-peer) was the primary independent variable. Measurement follow-up time (namely, baseline, mid-intervention, and post-intervention) was included along with its interaction with methodology group. Contrast statements were used for *post hoc* comparisons of the changes between the three methodology groups at different follow-ups. A random intercept was included to account for within patient correlations. Least Squares Means were obtained from these models to estimate changes in the total patient activation and lupus self-efficacy scores independently in each methodology. Tukey-Kramer adjustment was used for *post hoc* comparisons. Adjusted model included demographic variables, namely age, education, employment, and insurance. The parent study was powered for the primary outcome of change in health related quality of life (HRQOL) between baseline and 12 months post intervention. The minimum sample size was based on detecting a clinically meaningful difference of 0.35 standard deviation units (medium effect) based on prior studies (Chung et al., 2013; Keefe et al., 2013; Collings et al., 2014; Lu et al., 2014; Williams et al., 2014, 2018; Merianos et al., 2016). In this exploratory analysis estimates of the means for groups and group differences are provided along with confidence intervals and *p*-values. However, *p*-values should be interpreted with caution because the study is not specifically powered to test the corresponding hypotheses.

## Peer-To-Peer Results

Table 1 presents the general characteristics of the participants that utilized the three patient intervention methodologies of patient navigator, support group, and peer-to-peer. Participants in the patient navigator group were slightly older than those in the support group and peer-to-peer group with the median ages being between 45–54 and 35–44, respectively. About 43% of those in the patient navigator group were college graduates, whereas the median education level for the support group and peer-to-peer group was 17%. Marriage rates were similar across the methodology groups while unemployment rates were highest amongst the support group participants at 46.3%. Most of the participants were uninsured with rates spanning from 75.6% in the support group to 87.5% in the peer-to-peer group. The baseline patient activation scores were similar across the methodology groups with a mean of 30.79 in the patient navigator group, 33.56 in the support group, and 32.38 in the peer-to-peer group. Coping or self-efficacy scores were also similar across groups with means ranging from 327.06 to 359.86. The correlation (overall) between the two outcomes was 0.37, *p*-value < 0.0001.

**TABLE 1:** Demographic characteristics of participants in patient navigator, support group, and peer to peer intervention studies.

	Patient navigator (CALLS experimental)	Support group (PALS control)	Peer-to-Peer (PALS experimental)
*N*	14	52	48
Age (%)			
<25	3 (21.4)	4 (9.8)	5 (10.4)
25–34	2 (14.3)	15 (36.6)	13 (27.1)
35–44	2 (14.3)	9 (22.0)	18 (37.5)
45–54	5 (35.7)	7 (17.1)	6 (12.5)
55–64	1 (7.1)	6 (14.6)	4 (8.3)
≥65	1 (7.1)	0 (0.0)	2 (4.2)
Education ^*c*–^(%)			
<High School	0 (0.0)	1 (2.4)	7 (14.9)
High School	1 (7.1)	0 (0.0)	0 (0.0)
Some College	0 (0.0)	7 (17.1)	8 (17.0)
College Grad	6 (42.9)	0 (0.0)	0 (0.0)
Marriage = Other (%)	4 (28.6)	14 (34.1)	17 (36.2)
Unemployed (%)	3 (21.4)	19 (46.3)	15 (31.9)
Not insured (%)	11 (78.6)	31 (75.6)	42 (87.5)
Patient activation measure (PAM)			
Mean	30.79	33.56	32.38
(SD)	(3.40)	(5.01)	(3.71)
Total coping score			
Mean	359.86	367.78	327.06
(SD)	(128.11)	(113.93)	(133.68)

*^*c*^There were missing data for some participants.*

### Patient Activation Analysis

**FIGURE 1:**
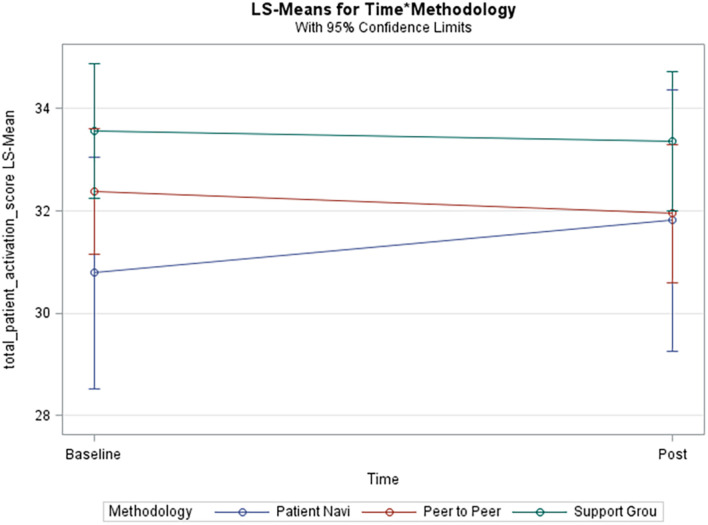
Unadjusted model of total patient activation score. Blue line (Patient Navi), patient navigator methodology; Red line (Peer to Peer), peer-to-peer methodology; Green line (Support Grou), support group methodology.

Table 2 represents results from this analysis. Means for methodology groups were statistically significantly different in terms of the total patient activation score (*p*-value 0.04). Figure 1, provides an interaction plot of the total patient activation scores over time for the different groups. The patient navigator method is seen to slightly increase over time although not statistically significant. The scores from the peer-to-peer method slightly decreased, while with the support group method, change was minimal. None of the pairwise comparisons *post hoc* were statistically significant as suggested by the overlapping confidence intervals in Figure 1. In the adjusted analysis the covariates age (*p*-value 0.01) and education (*p*-value 0.04) were statistically significantly associated with the total patient activation score. However, in terms of the methodology over time it did not alter statistical significance.

**TABLE 2:** Mean differences in total patient activation measure (PAM) from baseline to post-intervention time points between patient navigator, support group, and peer to peer intervention methodologies.

	Model 1^a^	Model 2^b^

Patient intervention methodology	Mean difference (95% CI); *P*-value	Mean difference (95% CI); *P*-value
Patient navigator (CALLS experimental only)	−1.03 (−4.44, 2.38); 0.55	−1.07 (−4.26, 2.12); 0.51
Support group (PALS control only)	0.20 (−1.69, 2.09); 0.83	0.28 (−1.52, 2.08); 0.76
Peer-to-Peer (PALS experimental only)	0.43 (−1.39, 2.25); 0.65	0.55 (−1.18, 2.27); 0.53
Patient navigator vs. support group (CALLS experimental vs. PALS control)	1.23 (−2.67, 5.14); 0.53	1.35 (−2.32, 5.02); 0.47
Patient navigator vs. peer-to-peer (CALLS experimental vs. PALS experimental)	1.46 (−2.41, 5.33); 0.46	1.62 (−2.02, 5.25); 0.38
Support group vs. peer-to-peer (PALS control vs. PALS experimental)	0.22 (−2.41, 2.85); 0.87	0.27 (−2.21, 2.75); 0.83

*^*a*^Unadjusted model.*

*^*b*^Model adjusts for age, education, employment, and insurance.*

*Means, C.I., and *p*-values are from a Linear Mixed Model adjusting for within patient correlations.*

### Coping (Lupus Self-Efficacy) Analysis

**FIGURE 2:**
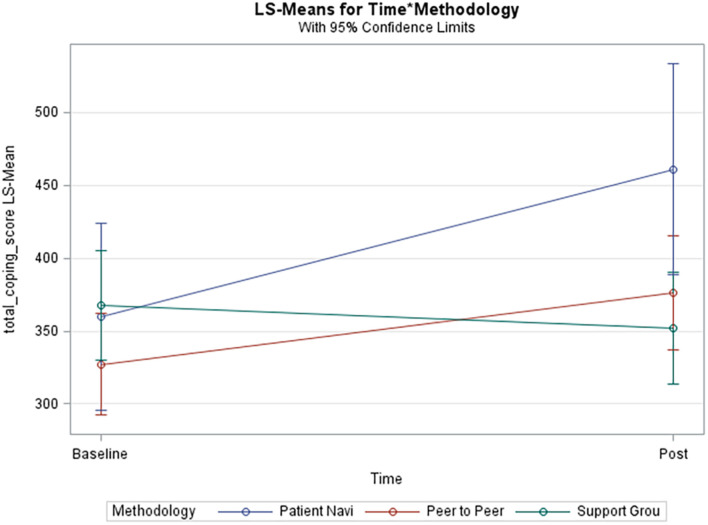
Unadjusted model of total coping score. Blue line (Patient Navi), patient navigator methodology; Red line (Peer to Peer), peer-to-peer methodology; Green line (Support Grou), support group methodology.

Table 3 represents results from this analysis. Data collection time points (*p*-value 0.03) were statistically significantly associated with total coping score. In Figure 2, the unadjusted model shows that total coping scores significantly increased over time in the patient navigator methodology group and insignificantly increased in the peer-to-peer group, but insignificantly decreased in the support group. The pairwise comparison between the patient navigator and support group methods showed a statistically significant difference in the unadjusted model (*p*-value 0.038), but not in the adjusted one (*p*-value 0.06). None of the other pairwise comparisons were statistically significantly associated with total coping scores.

**TABLE 3:** Differences in mean total coping score (Lupus Self-Efficacy) from baseline to post-intervention time points between patient navigator, support group, and peer to peer intervention methodologies.

	Model 1^a^	Model 2^b^

Patient intervention methodology	Mean difference (95% CI); *P*-value	LS means difference (95% CI); *P*-value
Patient navigator (CALLS experimental only)	−101.23 (−198.12, −4.35); 0.04	−98.78 (−194.63, −2.93); 0.04
Support group (PALS control only)	15.73 (−38.06, 69.52); 0.56	4.10 (−50.02, 58.22); 0.88
Peer-to-Peer (PALS experimental only)	−49.28 (−101.49, 2.94); 0.06	−46.08 (−98.26, 6.10); 0.08
Patient navigator vs. support group (CALLS experimental vs. PALS control)	116.96 (6.15, 227.8); 0.038	102.88 (−7.15, 212.91); 0.06
Patient navigator vs. peer-to-peer (CALLS experimental vs. PALS experimental)	51.9 (−58.1, 162.0); 0.35	52.70 (−56.68, 162.09); 0.34
Support group vs. peer-to-peer (PALS control vs. PALS experimental)	−65 (−139.9, 9.95); 0.09	−50.18 (−124.98, 24.63); 0.19

*^*a*^Unadjusted model.*

*^*b*^Model adjusts for age, education, employment, and insurance.*

## Discussion

Overall, this study set out to compare different patient intervention methodology groups: Patient navigator, support group and peer-to-peer when measuring patient activation and self-efficacy or coping among lupus patients. In addition to pharmaceutical therapies, socio-behavioral interventions have been shown to impact disease management. However, little is known about which socio-behavioral intervention methodology better supports SLE patients. Our research sought to compare three such methods and their respective impact on coping and patient activation. We focused on peer-to-peer pairings, a patient support group, and the use of a patient navigator. Additionally, we examined the differences of impact between models that were adjusted and unadjusted for the demographics of age, education, employment, and insurance. As such, we accounted for patients who may be more disadvantaged and have different and greater informational needs.

Our results indicate that there is a statistically significant relationship between patient activation and our intervention methodologies. Our results are somewhat consistent with previous studies that support the effectiveness of socio-behavioral interventions to increase activation (Parchman et al., 2010; Hibbard and Greene, 2013; Shively et al., 2013). However, in our study the change in patient activation scores from baseline to post-intervention, for each methodology were not statistically significant. Likewise, there was not a significant difference between the three intervention methodologies when comparing the changes of patient activation. Our results differ with previous studies who reported statistically significant increases in activation scores with socio-behavioral interventions (Alegría et al., 2009; Druss et al., 2010; Frosch et al., 2010). A potential contributing factor for this difference is that we had notably smaller sample sizes. In considering our smaller sample sizes, our results were still able to show that age and education are important predictors to examine when evaluating patient activation.

**FIGURE 3:**
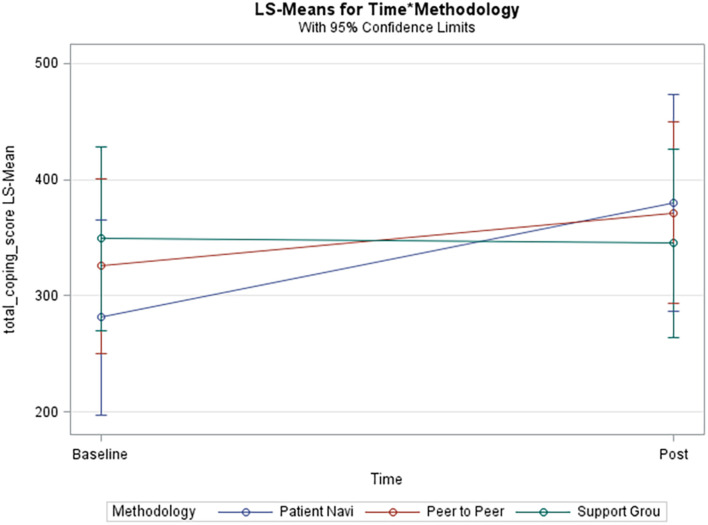
Adjusted model of total coping score. Blue line (Patient Navi), patient navigator methodology; Red line (Peer to Peer), peer-to-peer methodology; Green line (Support Grou), support group methodology.

Our results also indicate that there is a statistically significant relationship between coping and time, which consisted of 6 weeks and 12 weeks follow-ups (Figure 3). This is a crucial finding because one of the known barriers to patient self-efficacy is time, given that it can be overwhelming for a patient to receive and navigate through all their health education during their visit with the practitioner (Farley, 2020). The most significant improvement was seen among the patient navigator and peer-to-peer interventions over the follow-up time periods. This establishes a potential relationship between personalized disease coping methods and time. Relationships built amongst patients or between patients and a navigator strengthen over time and lead to improved coping self-efficacy. As a result, the need for a patient navigator becomes evident. In our study, the patient navigator methodology improved total coping scores with statistical significance even after adjusting for demographics. Higher coping scores are indicative of patients with greater lupus self-efficacy. Our results are consistent with previous studies that looked at the self-management of other chronic diseases and found that formal support systems and telephone interventions significantly promoted self-efficacy (Sohng, 2003; Farley, 2020). This is particularly important because self-efficacy or coping improves participation in health-promoting activities and treatment adherence (Farley, 2020).

Within our study there are some limitations. The main limitation is the lack of clinical data such as disease duration and disease activity (i.e., measurement of SLE disease activity before and after socio-behavioral interventions). Such measures were not consistently achieved across studies to be included, but should be considered in future research. Additionally, our SLE patients from these comparative studies may not be representative of all people with lupus. Their recruitment was limited to those who were hospitalized at MUSC generalizing to the broader United States or international SLE population tenuous. These patients may also have differing levels of travel burden and other needs than those not able to be hospitalized at MUSC, which may affect patient activation and coping. Again, our results should be carefully generalized to patients outside of South Carolina. The data were self-reported, so there is the potential for socially desirable responses and recall biases. Despite these limitations, the strengths of our study include the examination of intervention and control groups from two different research studies with beneficial results in a short timeframe of 12 months. The use of previously validated survey instruments in both studies allowed us to effectively measure patient activation and self-efficacy. From these measurements we were able to draw conclusions regarding the benefits of the socio-behavioral intervention methodologies used. Therefore, these data will be useful to guide policy and the allocation of resources to improve SLE patient outcomes across South Carolina and in similar states.

### Conclusion

Our results provide evidence to support the implementation of patient navigators for improving self-efficacy and to account for age and education when measuring patient activation (Parchman et al., 2010; Hibbard and Greene, 2013). As a result, patient navigators allow for sustainable behavior change that is crucial for high-risk populations to avoid complications, prevent deterioration and maintain function (Farley, 2020). Tailored and personalized interventions require a significant time investment from the patient and care team member but yield improvements in perceived coping which are not sufficiently addressed in standard clinical treatments. The majority of SLE patients that participated in our study were African American females, which is expected because lupus disproportionately impacts African American women. Therefore, our results suggest the need for additional research to further confirm that the patient navigator methodology is most effective for this specific patient population and whether it is suitable to be applied more broadly. This research is necessary to improve support for patient navigators, who may be part of a critical pathway toward improving disease self-management which could result in improved outcomes and reduced disease activity among SLE patients.

## Data Availability Statement

The raw data supporting the conclusions of this article will be made available by the authors, without undue reservation.

## Ethics Statement

The studies involving human participants were reviewed and approved by the Medical University of South Carolina Institutional Review Board. The patients/participants provided their written informed consent to participate in this study.

## Author Contributions

EW was the principal investigator. JO and LE were senior investigators. AW, HJ, and JR were involved in intervention development, implementation, evaluation, data analysis, and manuscript writing. AL provided content expertise and manuscript writing. CD-G, AB, and TF were involved in data analysis and manuscript writing. VR provided statistical oversight. All authors read and approved the final version for publication.

## Conflict of Interest

The authors declare that the research was conducted in the absence of any commercial or financial relationships that could be construed as a potential conflict of interest.

## Publisher’s Note

All claims expressed in this article are solely those of the authors and do not necessarily represent those of their affiliated organizations, or those of the publisher, the editors and the reviewers. Any product that may be evaluated in this article, or claim that may be made by its manufacturer, is not guaranteed or endorsed by the publisher.

## Abbreviations

CALLS: care-coordination approach to learning lupus self-management
LLDAS: Lupus Low Disease Activity State
MUSC: Medical University of South Carolina
PAM: patient activation measure
SAS: statistical analysis system
SLE: systemic lupus erythematosus.
