# Supplementary material for: Support Methodologies for African American Women With Lupus – Comparing Three Methods’ Effects on Patient Activation and Coping
Source: Front Psychol. 2021 Oct 5;12:734390. doi: 10.3389/fpsyg.2021.734390 (PMC8523887; doi:10.3389/fpsyg.2021.734390)
Supplement: Supplementary file 1 [file Data_Sheet_1.doc]

In the following questions, we would like to know how you feel about your ability to control your lupus symptoms. For each of the following questions, please circle the number which corresponds to the certainty that you can now perform the following activities or tasks.

________________________________________________________________________

1. How certain are you that you can control your fatigue?

0 10 20 30 40 50 60 70 80 90 100

very moderately very

uncertain uncertain certain

________________________________________________________________________

1. How certain are you that you can regulate your activity so as to be active without aggravating your lupus?

0 10 20 30 40 50 60 70 80 90 100

very moderately very

uncertain uncertain certain

________________________________________________________________________

1. How certain are you that you can do something to help yourself feel better if you are feeling blue?

0 10 20 30 40 50 60 70 80 90 100

very moderately very

uncertain uncertain certain

________________________________________________________________________

1. As compared with other people with lupus like yours, how certain are you that you can manage pain during your daily activities?

0 10 20 30 40 50 60 70 80 90 100

very moderately very

uncertain uncertain certain

________________________________________________________________________

1. How certain are you that you can manage your lupus symptoms so that you can do the things you enjoy doing?

0 10 20 30 40 50 60 70 80 90 100

very moderately very

uncertain uncertain certain

________________________________________________________________________

1. How certain are you that you can deal with the frustration of lupus?

0 10 20 30 40 50 60 70 80 90 100

very moderately very

uncertain uncertain certain
